# Association between high maternal haemoglobin levels in the first trimester and gestational diabetes mellitus: a prospective birth cohort study

**DOI:** 10.7189/jogh.16.04212

**Published:** 2026-07-10

**Authors:** Wei Li, Juan Yang, Haibo Li, Bin Sun, Zhengqin Wu, Haiyan Gao, Wenjuan Liu, Libo Xu, Yibing Zhu

**Affiliations:** 1 Fujian Obstetrics and Gynecology Hospital, Fuzhou, China; 2 Fujian Maternity and Child Health Hospital, College of Clinical Medicine for Obstetrics & Gynecology and Pediatrics, Fujian Medical University, Fuzhou, China; 3 Fujian Children's Hospital, Fuzhou, China

**Keywords:** haemoglobin, first trimester, gestational diabetes mellitus, cohort study

## Abstract

**Background:**

Recent studies suggest first-trimester maternal haemoglobin (Hb) may be linked to gestational diabetes mellitus (GDM), but evidence remains limited. This study examined the association between high first-trimester haemoglobin and GDM risk.

**Methods:**

This prospective study included 18 484 singleton pregnant women with a gestational age of ≤14 weeks in the Fujian Birth Cohort Study. Haemoglobin of the first trimester is divided into low (<110 g/L), normal (110–130 g/L) and high (≥130 g/L). Multivariate logistic regression and restricted cubic spline (RCS) models were used to explore the association between Hb level and GDM risk, and to gradually adjust sociodemographics, lifestyle and metabolic factors. Stratified and combined analysis was performed according to maternal age, pre-pregnancy body mass index and mode of conception.

**Results:**

Mean early Hb was 127.3 ± 9.6 g/L. The crude GDM incidence was 17.7%, 19.6%, and 25.3% in the low, normal, and high Hb groups, respectively. High Hb was significantly associated with increased GDM risk in crude (odds ratio (OR) = 1.40; 95% confidence interval (CI) = 1.30–1.50, *P* < 0.001), Model 2 (OR = 1.31; 95% CI = 1.22–1.41, *P* < 0.001), and Model 3 (OR = 1.24; 95% CI = 1.15–1.34, *P* < 0.001), with only 5.3% attenuation after adjusting for metabolic markers. Restricted cubic spline analyses showed a linear positive dose-response relationship between Hb and GDM risk (P for nonlinearity = 0.274). The association was stronger in non-advanced maternal age (non-AMA), overweight/obese, and spontaneously conceiving women, and combined effects were observed for high Hb with AMA, overweight/obesity, and assisted reproductive technology (ART).

**Conclusions:**

Early pregnancy haemoglobin is significantly associated with GDM risk, modified by age, pre-pregnancy BMI, and conception mode. Haemoglobin screening in early pregnancy may help identify high-risk women. Further studies are needed to clarify related mechanisms and evaluate interventions.

Gestational diabetes mellitus (GDM) affects about 14% of pregnancies around the world. This disease has shown a clear upward trend in low- and middle-income countries where people experience rapid changes in their nutritional habits [1,2]. The prevalence of GDM in China continues to rise. A meta-analysis based on IADPSG diagnostic criteria showed that the overall prevalence was 14.8%, and there were significant regional differences. In the economically developed eastern and southern regions, the prevalence rate is as high as 17.6–24.24%, while the northwest region is relatively low [2,3]. Gestational diabetes mellitus is associated with short-term adverse outcomes such as macrosomia, shoulder dystocia, birth trauma, premature delivery, and increased caesarean section rate, and may lead to long-term metabolic sequelae. Women who had GDM had a 10-fold higher risk of developing type 2 diabetes in the future. Their children are more likely to be obese or have abnormal glucose tolerance, and even face a higher risk of early-onset cardiovascular disease [4–6]. These cross-generational effects form a vicious circle of metabolic risk factors, leading to the continuous transmission of diabetes between generations. This situation highlights the importance of early identification and preventive intervention.

In recent years, an increasing number of studies have examined the potential role of haematological parameters, particularly Hb levels, in the pathophysiology of GDM [7]. Haemoglobin is measured universally in early pregnancy as part of routine antenatal anaemia screening. It is inexpensive, standardised, and available in virtually all healthcare settings globally. Haemoglobin is a key indicator of oxygen-carrying capacity, and its levels are influenced by iron homeostasis, plasma volume expansion, and erythropoiesis [8,9]. Evidence suggests that high haemoglobin concentrations may indicate inadequate plasma volume expansion or iron-catalysed oxidative stress, both of which are associated with impaired glucose tolerance [10]. However, clinical interpretation has traditionally focused on the low concentration range, while the prevention of anaemia has consistently remained a top priority on the public health agenda. Furthermore, the interaction between high haemoglobin levels in the first trimester and pre-pregnancy body mass index (BMI), maternal age, and mode of conception in the development of GDM is not yet fully understood.

Therefore, this study aims to investigate the relationship between maternal high haemoglobin levels in the first trimester and GDM in a large prospective cohort of Chinese pregnant women.

## METHODS

### Study population

The data of this study are based on the Fujian Provincial Birth Cohort Study (FJCCS), a prospective birth cohort study conducted in Fujian Maternal and Child Health Hospital. Participants were consecutively recruited from pregnant women with gestational age ≤14 weeks who received their first prenatal care at Fujian Maternal and Child Health Hospital and provided informed consent. During pregnancy, participants completed a standardised questionnaire and provided biological samples at three predefined time points: first trimester (≤14 weeks), second trimester (22–26 weeks), and third trimester (32–36 weeks). Detailed information about the queue design and overview is provided in the Supplementary Methods in the **Online Supplementary Document**. Data on maternal characteristics and medical information were collected by the electronic medical record and the questionnaires. This study was in accordance with the Declaration of Helsinki and approved by the Ethics Committee of Fujian Maternity and Child Health Hospital (Approval Number: 2017KR-030). All participants were informed and signed an informed consent form before participating [11].

**Figure 1:**
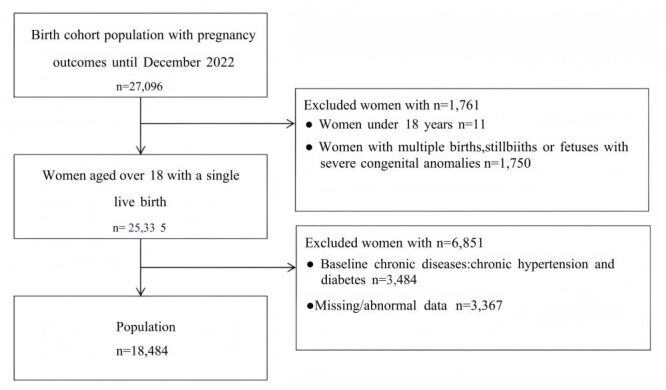
Flowchart of study population.

This study included singleton live births, defined as the delivery of one live-born infant from the current pregnancy, with complete physical examination and questionnaire data. This study excluded pregnant women under 18 years of age with chronic pre-pregnancy conditions (*e.g.* diabetes, hypertension), multiple pregnancies, or foetuses with major congenital anomalies (**Figure 1**).

### Data collection and variables

Data on maternal clinic characteristics was collected from electronic medical records, including maternal age, weight, height, parity, gravidity, mode of conception, diagnoses of diseases, and results of laboratory tests. Pre-pregnancy weight and height were recorded at the first prenatal visit based on both clinical examination and self-report; pre-pregnancy BMI was derived primarily from clinical records, supplemented by self-reported questionnaire data when necessary. Information on social factors such as maternal education level, ethnicity, passive smoking, pre-pregnancy drinking, and pre-pregnancy smoking was obtained through the questionnaire. The term ‘passive smoking exposure’ was defined as self-reported exposure to second-hand smoke at home and at work during the first three months of pregnancy.

According to international standards, maternal age was divided into two groups: the non-advanced maternal age (non-AMA, <35 years) and the advanced maternal age (AMA, ≥35 years) [12,13]. Pre-pregnancy BMI was calculated as weight (kg)/height (m)^2^. The pre-pregnancy BMI classification of participants was based on the following Chinese BMI classification standards: underweight group (UN, pre-pregnancy BMI<18.5 kg/m^2^), normal weight group (NW, 18.5 kg/m^2^≤pre-pregnancy BMI<24 kg/m^2^), overweight and obese group (OW & OB, pre-pregnancy BMI≥24.0 kg/m^2^) [14,15].

According to the standards set by the World Health Organization (WHO), a haemoglobin concentration of less than 110 g/L during pregnancy was classified as gestational anaemia [16,17]. There is currently no consensus on the precise threshold for high haemoglobin levels during pregnancy. This study applied clinical research standards to classify the proposed cut-off into three categories: low Hb group (<110 g/L), normal Hb group (110 g/L≤Hb ≤130 g/L), and high Hb group (≥130 g/L) [18,19].

GDM was diagnosed using a 75-g oral glucose tolerance test (OGTT) performed at 24–28 weeks of gestation (second trimester). A diagnosis of GDM is made if any of the following thresholds are reached or exceeded: fasting plasma glucose (FPG)≥5.1 mmol/L, 1-hour postprandial blood glucose ≥10.0 mmol/L, or 2-hour postprandial blood glucose ≥8.5 mmol/L [6,20].

Covariates were selected based on prior knowledge and clinical relevance [21,22]. Two sets of adjustment models were defined to clarify different aspects of the Hb-GDM association. Model 2 was adjusted according to the established demographic and lifestyle covariates: maternal age, race, pre-pregnancy BMI, gravidity, parity, education level, pre-pregnancy smoking, passive smoking, pre-pregnancy drinking, and mode of conception. If these covariates are primarily confounders rather than mediators, this model may approximate the total effect of haemoglobin on GDM risk. Model 3 further adjusted early metabolic markers [systolic blood pressure (SBP), FPG, triglyceride (TG), total cholesterol (TC)] in the first trimester. Since haemoglobin and these markers are measured simultaneously, their causal order is uncertain. These variables may play a partial mediating role in causality. Therefore, the model provides a conservative estimate of the Hb-GDM association independent of concurrent metabolic status, rather than a formal direct effect estimate.

### Statistical analysis

Continuous variables with normal distribution were expressed as mean ± standard deviation (SD), and Student *t* test was used to compare the two groups, or one-way analysis of variance was used to compare the three groups. Non-normally distributed variables were presented as median (interquartile range (IQR)) and analysed using the Mann-Whitney U test or Kruskal-Wallis test. Categorical variables are expressed as frequencies (percentages) and compared using the χ^2^ test. Fisher 's exact test is used when the expected cell count is less than 5.

Based on the normal haemoglobin group, three sequential multivariate logistic regression models were constructed:

1) Model 1: crude association (unadjusted);

2) Model 2: adjusted for non-metabolic confounding factors (maternal age, race, pre-pregnancy BMI, gravidity, parity, education level, pre-pregnancy smoking, passive smoking, pre-pregnancy drinking, and mode of conception), representing associations independent of demographic and lifestyle factors;

3) Model 3: additional adjustments were made to metabolic markers (SBP, FPG, TG, TC) in early pregnancy. Since these markers are measured simultaneously with haemoglobin, the causal order is uncertain. These variables can act as partial mediators. The model provides a conservative estimate of the Hb-GDM association independent of concurrent metabolic disorders, rather than a formal direct effect estimate, recognising that these factors may partially mediate this association. Odds ratio (OR) and 95% confidence interval (CI) were calculated to assess the independent association between haemoglobin category and GDM risk. Since the adjustment of the potential medium may slightly weaken the effect estimation and introduce the theoretical collider bias, the effect estimation of Model 3 should be interpreted as conservative.

Stratified analysis was performed according to maternal age (AMA/non-AMA), pre-pregnancy BMI category and mode of conception to estimate the association between haemoglobin and GDM in specific subgroups. Stratified analysis was conducted according to maternal age (AMA/non-AMA), pre-pregnancy BMI category and conception mode. The normal haemoglobin group in the corresponding group was used as the reference group in each layer. Combined effects and effect modification were evaluated by constructing joint categorical variables with the lowest-risk group as reference and including multiplicative interaction terms in the full models. The combined stratification analysis was also stratified by maternal age (AMA/non-AMA), pre-pregnancy BMI category and conception method, but the subgroup with the lowest risk of each stratification factor was uniformly selected as the common reference group. For example, the maternal age layer was based on the non-AMA and normal haemoglobin group, the pre-pregnancy BMI layer was based on the pre-pregnancy underweight and normal weight BMI and normal haemoglobin group, and the mode of conception layer was based on the spontaneous conception and normal haemoglobin group.

We conducted a restricted cubic spline (RCS) model to develop smooth curves to examine the possible nonlinear dose-response associations between Hb and GDM among the overall population and across subgroups. In these models, Hb was used as a continuous variable with three knots, and nonlinearity was tested using a likelihood ratio test, three nodes for the RCS were selected based on sample distribution and visual inspection.

A two-sided *P*-value <0.05 was considered statistically significant, and the analyses were performed using SPSS Statistics, version 25 (IBM Corp., Armonk, NY, USA) and *R* software, version 4.2.2 (R Foundation for Statistical Computing, Vienna, Austria).

## RESULTS

### Descriptive characteristics

A total of 18 484 pregnant women were included in this study, with an average haemoglobin level of 127.3 ± 9.6 g/L during the first trimester. Based on haemoglobin concentration, the women were divided into three groups: low (n = 730), normal (n = 10 729), and high (n = 7025) haemoglobin. Relevant baseline information is detailed in **Table 1**. The mean age of the pregnant women enrolled in the study was 30.3 ± 4.0 years, and the mean pre-pregnancy BMI was 21.2 ± 2.9 kg/m^2^. The study population primarily consisted of Han Chinese women with a relatively high overall educational level, with 15.0% of pregnant women being overweight or obese prior to pregnancy. Statistically significant differences were observed among the three groups in baseline characteristics such as parity, gravidity, and mode of conception, with all *P*-values less than 0.05. The mean systolic blood pressure in early pregnancy for the overall study population was 114.8 ± 10.8 mm Hg. Significant differences were also observed in various laboratory test indicators across the three haemoglobin groups. Furthermore, pregnant women in the high-haemoglobin group generally had relatively higher levels of fasting blood glucose, triglycerides, and total cholesterol than those in the low-haemoglobin group.

**Table 1:** Baseline characteristics stratified by maternal haemoglobin levels in first trimester*

Maternal characteristics	Total	Low Hb	Normal Hb	High Hb	*P* value
	**N = 18 484**	**n = 730**	**n = 10 729**	**n = 7025**	
**Demographic characteristics**					
Maternal age, years	30.2 ± 4.0	30.5 ± 4.0	30.3 ± 4.0	30.1 ± 4.0	<0.001
*Non-AMA*	16,257 (88.0)	623 (85.3)	6426 (87.9)	6208 (88.4)	0.051
*AMA*	2227 (12.0)	107 (14.7)	1303 (12.1)	817 (11.6)	
Ethnicity					
*Han*	18,092 (97.9)	713 (97.7)	10,480 (97.7)	6899 (98.2)	0.054
*Others*	392 (2.1)	17 (2.3)	249 (2.3)	126 (1.8)	
Education level					0.035
*Primary school and below*	50 (0.3)	5 (0.7)	27 (0.3)	18 (0.3)	
*Junior high school*	1576 (8.5)	79 (10.8)	891 (8.3)	606 (8.6)	
*High school*	2479 (13.4)	94 (12.9)	1440 (13.4)	945 (13.5)	
*Undergraduate*	13,104 (70.9)	510 (69.9)	7589 (70.7)	5005 (71.2)	
*Postgraduate and above*	1275 (6.9)	42 (5.8)	782 (7.3)	451 (6.4)	
**Lifestyle characteristics**					
Pre-pregnancy smoking					0.638
*Yes*	364 (2.0)	12 (1.6)	219 (2.0)	133 (1.9)	
*No*	18,120 (98.0)	718 (98.4)	10,510 (98.0)	6892 (98.1)	
Passive smoking					0.429
*Yes*	2794 (15.1)	108 (14.8)	1653 (15.4)	1033 (14.7)	
*No*	15,690 (84.9)	622 (85.2)	9076 (84.6)	5992 (85.3)	
Pre-pregnancy drinking					0.058
*Yes*	2371 (12.8)	94 (12.9)	1428 (13.3)	849 (12.1)	
*No*	16,113 (87.2)	636 (87.1)	9301 (86.7)	6176 (87.9)	
**Obstetric history**					
Parity					<0.001
*Nulliparous*	11,049 (59.8)	383 (52.5)	6217 (57.9)	4449 (63.3)	
*Parous*	7435 (40.2)	347 (47.5)	4512 (42.1)	2576 (36.7)	
Gravidity					<0.001
*1*	8085 (43.7)	284 (38.9)	4569 (42.6)	3232 (46.0)	
*2*	5575 (30.2)	228 (31.2)	3290 (30.7)	2057 (29.3)	
*≥3*	4824 (26.1)	218 (29.9)	2870 (26.7)	1736 (24.7)	
Mode of conception					<0.001
*Spontaneous*	17,193 (93.0)	691 (94.7)	10,047 (93.6)	6455 (91.9)	
*Assisted*	1291 (7.0)	39 (5.3)	682 (6.4)	570 (8.1)	
**Physical examination**					
Pre-pregnancy BMI, kg/m^2^	21.2 ± 2.9	20.4 ± 2.4	20.9 ± 2.7	21.7 ± 3.1	<0.001
*UW*	2839 (15.4)	141 (19.3)	1791 (16.7)	907 (12.9)	<0.001
*NW*	12,877 (69.7)	542 (74.2)	7665 (71.4)	4670 (66.5)	
*OW & OB*	2768 (15.0)	47 (6.4)	1273 (11.9)	1448 (20.6)	
First-trimester SBP, mmHg	114.8 ± 10.8	112.4 ± 11.4	113.9 ± 10.7	116.4 ± 10.7	<0.001
**Laboratory indicators**					
First-trimester Hb, g/L	127.3 ± 9.6	102.6 ± 6.8	123.0 ± 5.2	136.5 ± 4.7	<0.001
First-trimester FPG, mmol/L	4.7 ± 0.4	4.7 ± 0.3	4.7 ± 0.4	4.8 ± 0.4	<0.001
First-trimester TG, mmol/L	1.3 (1.0–1.6)	1.2 (1.0–1.5)	1.3 (1.0–1.6)	1.3 (1.1–1.6)	<0.001
First-trimester TC, mmol/L	4.7 ± 0.7	4.4 ± 0.8	4.6 ± 0.7	4.7 ± 0.7	<0.001

AMA – advanced maternal age, BMI – body mass index, FPG – fasting plasma glucose, Hb – haemoglobin, NW – normal weight, OB – obese, OW – overweight, SBP – systolic blood pressure, TC – total cholesterol, TG – triglyceride, UW – underweight

*Data are presented as mean ± standard deviation (SD) for continuous variables and n (%) for categorical variables.

### Relationship between maternal haemoglobin levels in the first trimester and GDM

**Table 2** presents the association between first-trimester haemoglobin levels and GDM across three models with distinct adjustment strategies. The crude incidence of GDM was lowest in the low Hb group (17.7%, n/N = 129/730) and highest in the high Hb group (25.3%, n/N = 1780/7,025), with the normal Hb group as reference (19.6%, n/N = 2099/10,729).

**Table 2:** Logistic regression analysis of the relationship between maternal haemoglobin levels in the first trimester and GDM

Group	Total	GDM, n (%)	Model 1		Model 2*		Model 3†	
			**Crude OR (95% CI)**	***P* value**	**OR (95% CI)**	***P* value**	**OR (95% CI^)^**	***P* value**
Low Hb	730	129 (17.7)	0.88 (0.73–1.07)	0.211	0.92 (0.75–1.12)	0.42	0.99 (0.81–1.21)	0.933
Normal Hb	10,729	2099 (19.6)	1 (Ref)		1 (Ref)		1 (Ref)	
High Hb	7,025	1780 (25.3)	1.40 (1.30–1.50)	<0.001	1.31 (1.22–1.41)	<0.001	1.24 (1.15–1.34)	<0.001

BMI – body mass index, CI – confidence interval, FPG – fasting plasma glucose, GDM – gestational diabetes mellitus, Hb – haemoglobin, OR – odds ratio, SBP – systolic blood pressure, TC – total cholesterol, TG – triglyceride

*Model 2 adjusted: maternal age, ethnicity, pre-pregnancy BMI, gravidity, parity, education level, pre-pregnancy smoking, passive smoking, pre-pregnancy drinking, and conception mode.

†Model 3 adjusted: Model 2 + first-trimester SBP, first-trimester FPG, first-trimester TG, first-trimester TC.

In the unadjusted analysis, high haemoglobin levels were found to be significantly associated with an increased risk of GDM (crude OR = 1.40; 95% CI = 1.30–1.50, *P* < 0.001), while low haemoglobin levels were not associated with an increased risk of GDM (crude OR = 0.88; 95% CI = 0.73–1.07, *P* = 0.211). After adjusting for non-metabolic confounding factors, including maternal age, race, pre-pregnancy BMI, gravidity, parity, education level, pre-pregnancy smoking, passive smoking, pre-pregnancy drinking, and mode of conception (Model 2), the model estimated the association between haemoglobin and GDM, regardless of demographic and lifestyle factors. If these covariates are primarily confounders rather than mediators, this model may approximate the total effect of haemoglobin on GDM risk, and high haemoglobin was still significantly associated with GDM (adjusted OR = 1.31; 95% CI = 1.22–1.41, *P* < 0.001). In Model 3, systolic blood pressure, fasting blood glucose, triglyceride, and total cholesterol in the first trimester were further adjusted. Since haemoglobin and these metabolic markers were measured simultaneously, their causal order was uncertain. These variables may play a partial mediating role in causality. Therefore, the model provided a conservative estimate of the Hb-GDM association independent of concurrent metabolic status, rather than a formal direct effect estimate. Although this association was moderately weakened, it was still statistically significant (adjusted OR = 1.24; 95% CI = 1.15–1.34, *P* < 0.001). The 5.3% relative attenuation from Model 2 to Model 3 indicated that early metabolic markers account for only a small part of the Hb-GDM association. Low haemoglobin showed no significant association with GDM in crude, Model 2, or Model 3 analyses.

### Stratified analysis by maternal characteristics

All stratified analyses were conducted using the same adjustment set as Model 3, providing conservative effect estimates adjusted for early metabolic markers. Compared to normal haemoglobin levels, high haemoglobin levels were associated with an adjusted OR of 1.14 (95% CI = 0.94–1.39, *P* = 0.175) in the AMA group, though this was not statistically significant after adjustment. Low haemoglobin levels showed no significant association (adjusted OR = 1.34; 95% CI = 0.88–2.05, *P* = 0.178). In contrast, high haemoglobin remained significantly associated with GDM among non-AMA women even after adjustment (adjusted OR = 1.24; 95% CI = 1.14–1.34, *P* < 0.001), whereas low haemoglobin was not (adjusted OR = 0.91; 95% CI = 0.73–1.15, *P* = 0.447).

For overweight and obese women, high haemoglobin levels significantly increased the risk of GDM (adjusted OR = 1.31; 95% CI = 1.11–1.55, *P* = 0.002). The same association was observed in women with normal weight (adjusted OR = 1.27; 95% CI = 1.16–1.39, *P* < 0.001). The study also found that this significant association was not seen in underweight women regardless of haemoglobin levels. In addition, the increased risk of GDM associated with high haemoglobin levels was most pronounced in overweight and obese women, indicating an interaction between haemoglobin levels and these two factors.

The mode of conception also influenced the relationship. Women who conceived spontaneously showed a positive association between high haemoglobin levels and GDM (adjusted OR = 1.25; 95% CI = 1.15–1.35, *P* < 0.001), whereas those who underwent assisted reproduction showed no significant association (adjusted OR = 1.15; 95% CI = 0.89 – 1.47, *P* = 0.293). The test for interaction was significant (*P* < 0.001), indicating that the effect of haemoglobin on GDM differs significantly by mode of conception.

### Combined stratification analyses

It should be emphasised that the reference group and analytical purpose differ between **Table 3** and Table S1 in the **Online Supplementary Document**. Combined stratification analyses were also adjusted according to the Model 3 framework. Using non-AMA women with normal haemoglobin levels as a common reference group (Table S1 in the **Online Supplementary Document****)**, AMA women with higher haemoglobin levels had a significantly higher risk of GDM (adjusted OR = 1.81; 95% CI = 1.53–2.13, *P* < 0.001). The estimated combined effect differed from the stratification-specific association (OR = 1.14; 95% CI = 0.94–1.39, *P* = 0.175) (**Table 3**).

**Table 3:** Stratified analysis of the association between maternal haemoglobin levels in the first trimester and GDM*

Variable	Low Hb					Normal Hb			High Hb					*P* for interaction
	**GDM, n (%)**	**Crude**		**Adjusted**		**GDM, n (%)**	**Crude**	**Adjusted**	**GDM, n (%)**	**Crude**		**Adjusted**		
		**OR (95% CI)**	***P-*value**	**OR (95% CI)**	***P-*value**		**OR (95% CI)**	**OR (95% CI)**		**OR (95% CI)**	***P-*value**	**OR (95% CI)**	***P-*value**	
Maternal age														<0.001
*Non-AMA*	92 (14.8)	0.79 (0.63–0.99)	0.044	0.91 (0.73–1.15)	0.447	1693 (18.0)	1 (Ref)	1 (Ref)	1482 (23.9)	1.43 (1.32–1.55)	<0.001	1.24 (1.14–1.34)	<0.001	
*AMA*	37 (34.6)	1.17 (0.77–1.77)	0.464	1.34 (0.88–2.05)	0.178	406 (31.2)	1 (Ref)	1 (Ref)	298 (36.5)	1.27 (1.06–1.53)	0.011	1.14 (0.94–1.39)	0.175	
Pre-pregnancy BMI														0.011
*UW*	18 (12.8)	0.89 (0.53–1.49)	0.654	0.90 (0.53–1.50)	0.677	253 (14.1)	1 (Ref)	1 (Ref)	137 (15.1)	1.08 (0.86–1.36)	0.495	1.03 (0.82–1.29)	0.812	
*NW*	102 (18.8)	0.98 (0.79–1.23)	0.878	1.05 (0.84–1.32)	0.673	1463 (19.1)	1 (Ref)	1 (Ref)	1117 (23.9)	1.33 (1.22–1.46)	<0.001	1.27 (1.16–1.39)	<0.001	
*OW & OB*	9 (19.1)	0.55 (0.26–1.15)	0.112	0.60 (0.28–1.27)	0.179	383 (30.1)	1 (Ref)	1 (Ref)	526 (36.3)	1.33 (1.13–1.56)	0.001	1.31 (1.11–1.55)	0.002	
Mode of conception														<0.001
*Spontaneous*	123 (17.8)	0.93 (0.76–1.13)	0.451	1.02 (0.83–1.26)	0.833	1905 (19.0)	1 (Ref)	1 (Ref)	1596 (24.7)	1.40 (1.30–1.51)	<0.001	1.25 (1.15–1.35)	<0.001	
*Assisted*	6 (15.4)	0.46 (0.19–1.11)	0.083	0.59 (0.24–1.48)	0.263	194 (28.4)	1 (Ref)	1 (Ref)	184 (32.3)	1.20 (0.94–1.53)	0.141	1.15 (0.89–1.47)	0.293	

AMA – advanced maternal age, BMI – body mass index, Hb – haemoglobin, NW – normal weight, OB – obese, OW – overweight, UW – underweight

*Adjusted for the same covariates as Model 3 in **Table 2**, except the stratification variable itself.

Two key differences explain the distinct findings. First, **Table 3** compares high haemoglobin *vs.* normal haemoglobin within the AMA subgroup, whereas Table S1 in the **Online Supplementary Document** compares AMA women with high haemoglobin to non-AMA women with normal haemoglobin (the lowest-risk reference group). Second, in **Table 3**, BMI was included as a covariate, whereas in Table S1 in the **Online Supplementary Document**, age and BMI were incorporated as part of the combined stratification groups. The significant combined effect in Table S1 in the **Online Supplementary Document** reflects the combined, cumulative risk of advanced maternal age and high haemoglobin, rather than the independent effect of haemoglobin alone among AMA women. The significant combined effect showed that compared with the lowest risk reference group, the AMA women and high haemoglobin had a combined, cumulative effect, which together increased the risk of GDM, rather than reflecting the independent effect of haemoglobin in the AMA women.

Of the BMI-Hb combinations, overweight and obese (OW&OB) women with high haemoglobin had the highest risk (adjusted OR = 1.80; 95% CI = 1.59–2.05, *P* < 0.001). Even among women with underweight or normal pre-pregnancy BMI, high haemoglobin levels were associated with a significant 24% higher odds of GDM (adjusted OR = 1.24; 95% CI = 1.14–1.35, *P* < 0.001). Although there was no significant elevation in risk for underweight women with low haemoglobin (adjusted OR = 1.03; 95% CI = 0.83–1.27, *P* = 0.807), the consistent pattern across BMI strata highlights the fact that increased haemoglobin has an independent contribution to GDM risk across different BMI groups.

Women who conceived spontaneously and had high haemoglobin levels were at significantly greater risk of GDM than those who conceived spontaneously with normal haemoglobin levels (adjusted OR = 1.25; 95% CI = 1.16–1.36, *P* < 0.001). Similarly, women undergoing assisted reproduction who had high haemoglobin levels were also at significantly greater risk than the same reference group (adjusted OR = 1.34; 95% CI = 1.10–1.64, *P* = 0.003).

### Nonlinear dose-response relationships

**Figure 2:**
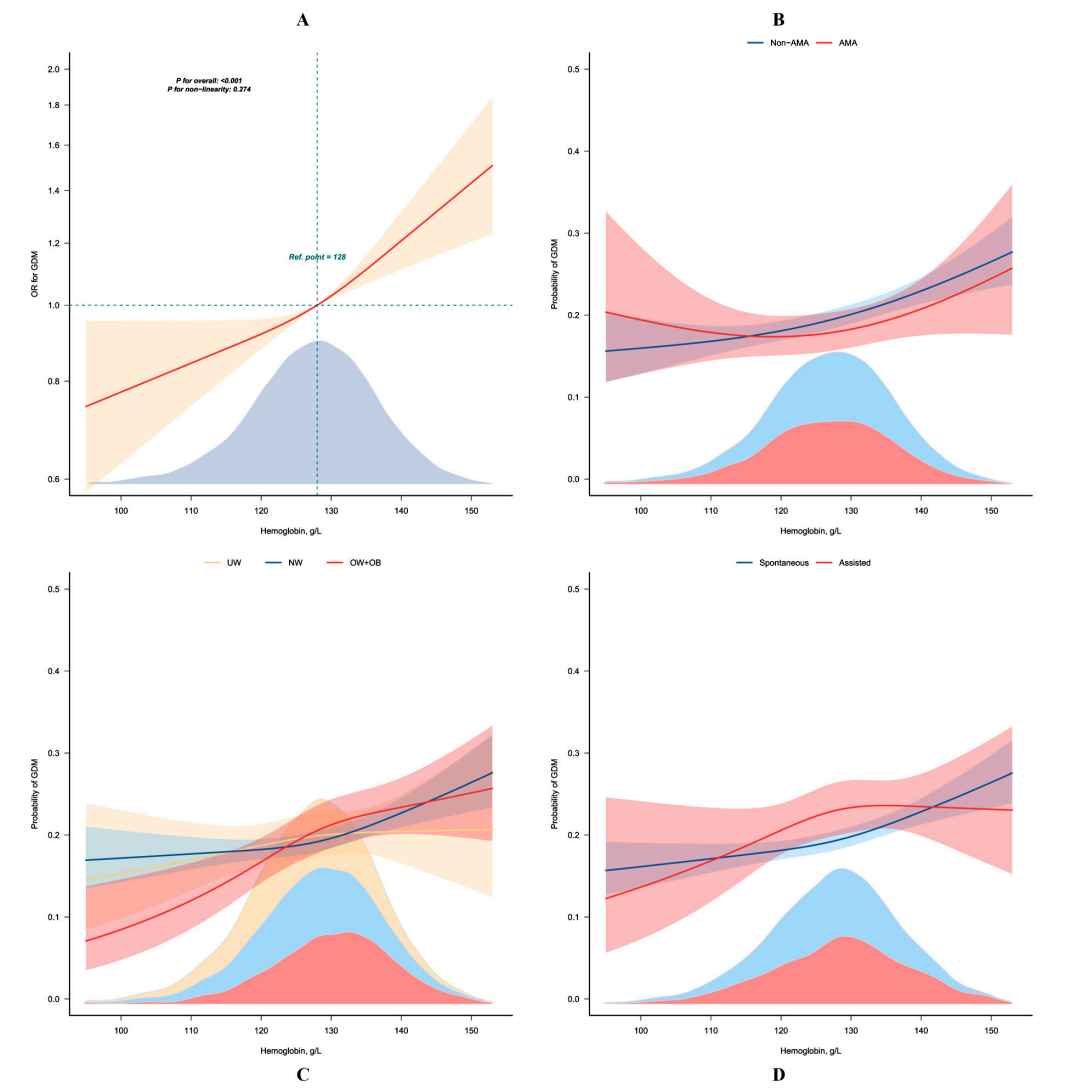
Restricted cubic spline analysis of the association between first-trimester maternal haemoglobin levels and GDM risk. **Panel A.** Overall association. **Panel B.** Subgroup analysis by maternal age. **Panel C.** Subgroup analysis by pre-pregnancy BMI. **Panel D.** Subgroup analysis by mode of conception. Adjusted for the same covariates as Model 3 in **Table 2**, except the stratification variable itself. AMA – advanced maternal age, BMI – body mass index, GDM – gestational diabetes mellitus, NW – normal weight, OB – obese, OW – overweight, UW – underweight.

We used a RCS model to analyse the nonlinear association between haemoglobin concentration and the risk of GDM. After adjustment according to the Model 3 framework (adjusted for non-metabolic confounders plus early metabolic markers), haemoglobin concentration was significantly and linearly associated with the risk of GDM (*P* < 0.001), with no significant nonlinearity observed (*P* for nonlinearity test = 0.274) (**Figure 2**, Panel A). Using a haemoglobin concentration of 128 g/L as the reference point, the risk of GDM decreased when haemoglobin concentration was below this level (OR<1.0), while the risk continued to rise when it was above this level, with an OR of approximately 1.5 (upper 95% CI approximately 1.8) at 150 g/L.

When stratified by age, both the non-AMA and AMA groups showed a trend of increasing GDM probability with rising haemoglobin levels, however, the confidence intervals for the AMA group were wider, suggesting greater variability or a smaller sample size in the AMA population (**Figure 2**, Panel). Additionally, when stratified by pre-pregnancy BMI, among the underweight, normal weight, and overweight/obese groups, the overweight/obese group already had a higher baseline probability of GDM at low haemoglobin levels (approximately 0.07–0.08) and exhibited the steepest upward trend as haemoglobin levels increased, suggesting a potential combined effect between high haemoglobin and obesity (**Figure 2****,** Panel C). Finally, when stratified by the mode of conception, the spontaneous and assisted reproduction groups exhibited similar patterns on the GDM probability curve. However, the density distribution in the assisted reproduction group was relatively higher in the low haemoglobin region (**Figure 2****,** Panel D).

## DISCUSSION

High haemoglobin levels are widely regarded as a marker of adequate nutritional status [23]. However, contrary to the traditional clinical emphasis on preventing anaemia, our findings suggest that high haemoglobin levels may reflect adverse physiological adaptations that predispose women to impaired glucose tolerance. Despite variations in ethnic and geographical backgrounds, the significant association observed in our study aligns with recent findings from other cohort studies [7,24,25]. However, there have also been conflicting research reports that found no significant association between high haemoglobin levels and GDM risk [26]. These differences may be attributed to the absence of early pathology of GDM, different screening schemes, and insufficient statistical efficacy. Alternatively, the relationship between haemoglobin and GDM might differ across ethnic groups due to population-specific factors, including varying baseline iron stores, genetic polymorphisms in iron metabolism genes, or distinct patterns of plasma volume expansion during pregnancy [26].

This study suggests a significant association between high haemoglobin levels in early pregnancy and GDM. From a mechanistic perspective, this may be attributed to physiological blood dilution failure. During a normal pregnancy, the volume of plasma in a woman's body increases. However, this expansion is disproportionate to the increase in red blood cell mass. It is this physiological change that leads to a natural decrease in haemoglobin concentration [17]. Conversely, if plasma volume expansion is insufficient, the body will experience a state of relative blood concentration. This condition is clinically manifested by higher haemoglobin levels in early pregnancy [10]. Young et al.'s and Jung et al.'s researches examined this phenomenon in depth. They highlighted that this state of insufficient plasma volume can have negative consequences. Specifically, it may impair the perfusion function of the uterus and placenta. Furthermore, it can lead to chronic oxidative stress reactions and mild inflammation in the body [27,28]. This high blood concentration and viscosity may exacerbate insulin resistance, significantly increasing the risk of GDM. Therefore, high haemoglobin levels occurring in early pregnancy should not be considered a random finding in laboratory testing, but rather a potential biomarker reflecting maternal hemodynamic maladjustment.

In our Model 3, we further adjusted the early pregnancy metabolic markers (FPG, TG, TC, SBP) that may play a partial mediating role in the causal pathway. The results showed that this association was still statistically significant. This provides a conservative estimate of the Hb-GDM association independent of concurrent metabolic status, but not a formal direct effect estimate. This finding supports the hypothesis that iron overload or changes in erythropoiesis may impair glucose homeostasis through a mechanism that is partially independent of concurrent metabolic disorders.

Additionally, we must consider the clinical significance of high haemoglobin levels. They may indicate excessive iron storage in pregnant women's bodies [23,29]. Recent systematic reviews and meta-analyses have provided important evidence. These studies suggest that excessive free iron can trigger the production of reactive oxygen species in the body. This process is primarily achieved through the Fenton reaction [10]. This reaction triggers a series of negative effects. For example, it can cause oxidative damage to pancreatic β-cells [30]. Meanwhile, the insulin secretion function of these cells is impaired. Abnormal iron accumulation is not limited to the pancreas. When iron accumulates in the liver and skeletal muscles, for example, it can also have a destructive effect. This interferes with the normal insulin receptor signalling process. This will further exacerbate the body's insulin resistance [24,25]. In this study, we adjusted for known confounding factors, yet this association persisted. This indicates that the relationship between iron metabolism, plasma volume dynamics, and glucose homeostasis is complex. From Model 2 to Model 3, OR decreased moderately by 5.3%, which supported that early metabolic factors accounted for only a small part of the Hb-GDM association, indicating that alternative pathways, such as direct iron-mediated oxidative stress, may play an important role.

Future research should therefore include direct measurement of iron status. Specific indicators to be measured should include ferritin and transferrin saturation. Only in this way can these physiological pathways be accurately elucidated. It is worth noting that the existing iron supplementation research provides important clues. Consistent evidence suggests that high levels of endogenous haemoglobin during pregnancy are significantly associated with adverse metabolic outcomes such as GDM. In addition, the increase of core iron metabolism markers such as serum ferritin will also independently increase the above adverse pregnancy and metabolic risks, which are mainly mediated by oxidative stress and insulin resistance [30,31].

This study provides us with new insights into the biological mechanism between haemoglobin and abnormal glucose tolerance. This study obtained these insights by observing the different effects that exist in different subgroups, and this information is also helpful for more targeted risk stratification. After full adjustment in the AMA subgroup, there was no significant association between high haemoglobin and GDM, which may be related to the attenuation effect of BMI and metabolic covariates and the lack of statistical efficiency in the subgroup. The comprehensive stratification results in Table S1 in the **Online Supplementary Document** suggest that advanced age and high haemoglobin have a cumulative risk effect, rather than the independent role of haemoglobin in the AMA population. Compared with non-AMA and low-risk women with normal haemoglobin, the risk of GDM in AMA combined with high haemoglobin is significantly higher, which supports that haemoglobin can be used for GDM risk stratification. Therefore, although high haemoglobin after adjustment for metabolic factors is not a strong independent predictor of GDM in women with AMA, the combined high-risk population still needs to strengthen GDM screening and monitoring. In addition, previous evidence has shown that higher haemoglobin levels in early pregnancy are significantly associated with pre-pregnancy overweight/obesity. Furthermore, high haemoglobin levels may reflect adequate iron stores and relatively sufficient iron intake (food or supplements), rather than being simply attributable to younger maternal age. Therefore, even among younger pregnant women, the clinical significance of elevated haemoglobin levels in early pregnancy should not be overlooked [24,32].

In the combined stratification analysis of pre-pregnancy BMI, we found that women who were overweight or obese and had higher haemoglobin levels constituted the highest risk group. This finding may be related to the interplay between pathological and physiological processes. Specifically, obesity is associated with chronic low-grade inflammation, which creates a unique internal environment that may enhance iron-catalysed free radical generation. Furthermore, this process may contribute to disproportionate pancreatic damage through increased oxidative stress [23,25].

The findings regarding the mode of conception were complex. In the ART subgroup, the association between high haemoglobin and GDM was not statistically significant (**Table 3**), which may be due to the limited sample size and the leading role of ART-related risk factors. This is consistent with the pattern observed in the AMA subgroup, where high haemoglobin also did not show a significant independent effect. However, in the comprehensive stratified analysis (Table S1 in the **Online Supplementary Document**), with non-AMA women with normal haemoglobin levels serving as a common low-risk reference group, women who conceived through ART and had high haemoglobin levels exhibited a significantly increased risk (OR = 1.34; 95% CI = 1.10, 1.64, *P* = 0.003). Similar to the findings of AMA, after full adjustment, high haemoglobin may not be a statistically significant independent predictor in the ART subgroup, but the comprehensive analysis identified a high-risk phenotype at the population level, which is worthy of strengthening clinical monitoring [33]. The emergence of significance in the combined stratification shows that the risk associated with high haemoglobin levels still exists, but in ART pregnancy greater statistical power is needed to detect this risk. Overall, these interactions underscore that the clinical value of high haemoglobin levels depends on the context. For example, we need to pay close attention to those pregnancies that are already high-risk, particularly women with spontaneous conception and high haemoglobin levels. On the other hand, for ART pregnancy, because the method itself has a higher baseline risk, it is necessary to conduct comprehensive metabolic monitoring regardless of the level of haemoglobin in pregnant women.

### Limitations

Several limitations of this study should be acknowledged. First, despite the prospective design ensuring that exposure assessment preceded the outcome, the observational nature of our study precludes definitive causal inference. Unlike randomised controlled trials, we cannot rule out the influence of unmeasured confounders such as specific dietary patterns, genetic polymorphisms affecting iron metabolism, or socioeconomic factors that may influence both haemoglobin levels and risk of diabetes [19,34]. It is worth noting that haemoglobin-related genetic variants, including α-thalassemia, β-thalassemia, and haemoglobin E (HbE), are very common in Fujian Province and may alter haemoglobin-GDM association by altering erythropoiesis, iron overload, or pancreatic β-cell dysfunction. These common genetic features were not measured in our study and may represent important unmeasured effect regulators [35–39]. Second, as this study represents a secondary analysis of the existing FJBCS data set, we lacked direct measurements of the underlying physiological drivers, particularly plasma volume expansion, blood viscosity, and iron stores (*e.g.* ferritin and transferrin saturation) [30,31]. Consequently, we used haemoglobin as a proxy marker. Although supported by existing literature, this indirect approach limits our ability to definitively determine whether the observed risk is primarily driven by haemoconcentration, iron overload, or both. Future mechanistic studies that incorporate these direct biomarkers are essential to clarify these pathways. Third, although our overall cohort is large, the number of women undergoing ART was relatively small compared to the number of women who conceived spontaneously. The limited sample size in the ART subgroup may have reduced statistical power to detect interactions within specific ART modalities, such as a comparison between *in vitro* fertilisation and intracytoplasmic sperm injection. These subgroup findings require validation in multicentre populations with larger ART sample sizes.

## CONCLUSIONS

In conclusion, increased first-trimester haemoglobin levels are a significant and independent predictor of GDM. This finding likely reflects failed physiological haemodilution and the subsequent metabolic stress. We therefore recommend including haemoglobin levels in early pregnancy risk management strategies. Identifying and addressing high levels of haemoglobin could enable clinicians to implement preventive measures promptly. This could ultimately improve maternal and perinatal outcomes in an era of rising GDM prevalence.

**Data availability:** Data are available on request from the corresponding author.

## Additional material


Online Supplementary Document
