# Supplementary material for: Association between high maternal haemoglobin levels in the first trimester and gestational diabetes mellitus: a prospective birth cohort study
Source: J Glob Health. 2026 Jul 10;16:04212. doi: 10.7189/jogh.16.04212 (PMC13351598; doi:10.7189/jogh.16.04212)
Supplement: Online Supplementary Document [file jogh-16-04212-s001.pdf]

Supplement to: Li W, Yang J, Li H, Sun B, Wu Z, Gao H, Liu W, Xu L, Zhu Y. Association between high maternal haemoglobin levels in the first trimester and gestational diabetes mellitus: a prospective birth cohort study. J Glob Health. 2026;16:04212.

Methods of the Fujian Birth Cohort Study (FJBCS)

The Fujian Birth Cohort Study (FJBCS) is a large-scale, prospective, ongoing birth cohort study conducted at Fujian Maternity and Child Health Hospital. It aims to investigate the associations between prenatal exposures and adverse pregnancy outcomes as well as long-term childhood health and development in southeastern China. The study was approved by the Ethics Committee of Fujian Maternity and Child Health Hospital (Approval No. 2017KR-030), and written informed consent was obtained from all participants.

1. Study Participants

Participants were pregnant women receiving their first prenatal care at Fujian Maternity and Child Health Hospital.

Inclusion criteria: gestational age ≤14 weeks, and eligible for complete follow-up and data collection.

Exclusion criteria: severe physical or mental disorders that may affect the study outcomes, including severe liver/kidney diseases, cerebrovascular diseases, mental illnesses, and intellectual disability.

2. Prenatal Follow-up and Data Collection

Three prenatal follow-up visits were scheduled, and all data were collected by uniformly trained nurses or investigators using standardized questionnaires and biological sample collection.

- **First trimester (≤14 weeks)**: Completed at the first prenatal visit in the obstetric clinic. After providing informed consent, participants finished questionnaires via dedicated tablet computers.

- **Second trimester (22–26 weeks)**: Questionnaires were mainly completed online via mobile devices; non-responders completed on-site questionnaires during prenatal visits.
- **Third trimester (32–36 weeks)**: The same as the second trimester, with online completion as the primary mode and on-site completion as a supplement.

Questionnaires covered sociodemographic characteristics, physical measurements, medical history, lifestyle, occupational exposure, nutrient supplementation, and other relevant information.

3. Biospecimen Collection and Management

Peripheral blood and urine samples were collected at each follow-up visit. Cord blood, amniotic fluid, placenta, and muscle tissue were additionally collected for birth defect cases and matched controls at delivery.

- First trimester: anticoagulated blood, non-anticoagulated blood, urine
- Second trimester: anticoagulated blood, non-anticoagulated blood
- Third trimester: anticoagulated blood, non-anticoagulated blood
- Delivery: cord blood, amniotic fluid, placenta, muscle tissue

All biospecimens were processed, aliquoted, and stored at low temperatures by professional staff of the hospital biobank, which has been approved for national human genetic resource conservation. Subsequent testing projects were determined after thorough discussion by the research group and based on previous studies to ensure the scientificity and rationality of biospecimen utilization.

Table S1. A combined stratification analysis of maternal characteristics and hemoglobin levels in the first trimester of GDM*

Combination group	GDM, n (%)	Adjusted OR (95% CI)	*P* value
Maternal age x Hb combination			
Non-AMA & Low Hb	92 (14.8)	0.91 (0.72-1.14)	0.41
Non-AMA & Normal Hb	1,693 (18.0)	1 (Ref)	
Non-AMA & High Hb	1,482 (23.9)	1.24 (1.15-1.35)	<0.001
AMA & Low Hb	37 (34.6)	2.26 (1.50-3.43)	<0.001
AMA & Normal Hb	406 (31.2)	1.63 (1.42-1.87)	<0.001
AMA & High Hb	298 (36.5)	1.81 (1.53-2.13)	<0.001
Pre-pregnancy BMI x Hb combination			
UW + NW & Low Hb	120 (17.6)	1.03 (0.83-1.27)	0.807
UW + NW & Normal Hb	1,716 (18.1)	1 (Ref)	
UW + NW & High Hb	1,254 (22.5)	1.24 (1.14-1.35)	<0.001
OW + OB & Low Hb	9 (19.1)	0.79 (0.37-1.66)	0.534

Combination group	GDM, n (%)	Adjusted OR (95% CI)	*P* value
OW + OB & Normal Hb	383 (30.1)	1.37 (1.19-1.57)	<0.001
OW + OB & High Hb	526 (36.3)	1.80 (1.59-2.05)	<0.001
Mode of conception x Hb combination			
Spontaneous & Low Hb + Normal Hb	123 (17.8)	1.02 (0.83-1.25)	0.864
Spontaneous & Normal Hb	1,905 (19.0)	1 (Ref)	
Spontaneous & High Hb	1,596 (24.7)	1.25 (1.16-1.36)	<0.001
Assisted & Low Hb + Normal Hb	6 (15.4)	0.78 (0.32-1.90)	0.584
Assisted & Normal Hb	194 (28.4)	1.22 (1.01-1.47)	0.038
Assisted & High Hb	184 (32.3)	1.34 (1.10-1.64)	0.003

Hb - hemoglobin, AMA - advanced maternal age, BMI - body mass index, UW - underweight, NW - normal weight, OW - overweight, OB - obese.

* Adjusted for the same covariates as Model 3 in Table 2, except the stratification variable itself
